# Elevating CDCA3 Levels Enhances Tyrosine Kinase Inhibitor Sensitivity in TKI-Resistant EGFR Mutant Non-Small-Cell Lung Cancer

**DOI:** 10.3390/cancers13184651

**Published:** 2021-09-16

**Authors:** Katherine B. Sahin, Esha T. Shah, Genevieve P. Ferguson, Christopher Molloy, Priyakshi Kalita-de Croft, Sarah A. Hayes, Amanda Hudson, Emily Colvin, Hannah Kamitakahara, Rozelle Harvie, Csilla Hasovits, Tashbib Khan, Pascal H. G. Duijf, Viive M. Howell, Yaowu He, Emma Bolderson, John D. Hooper, Sunil R. Lakhani, Derek J. Richard, Kenneth J. O’Byrne, Mark N. Adams

**Affiliations:** 1 Centre for Genomics and Personalised Health, School of Biomedical Sciences, Faculty of Health, Queensland University of Technology, Brisbane, QLD 4059, Australia; Katherine.sahin@hdr.qut.edu.au (K.B.S.); e.shah@qut.edu.au (E.T.S.); genevieve.ferguson@hdr.qut.edu.au (G.P.F.); christopher.molloy@hdr.qut.edu.au (C.M.); pascal.duijf@qut.edu.au (P.H.G.D.); emma.bolderson@qut.edu.au (E.B.); derek.richard@qut.edu.au (D.J.R.); 2 UQ Centre for Clinical Research, Faculty of Medicine, University of Queensland, Herston, QLD 4006, Australia; p.kalita@uq.edu.au (P.K.-d.C.); s.lakhani@uq.edu.au (S.R.L.); 3 Bill Walsh Translational Research Laboratory, Faculty of Medicine and Health, Kolling Institute, University of Sydney, Royal North Shore Hospital, Reserve Road, St Leonards, NSW 2065, Australia; sarah.hayes@sydney.edu.au (S.A.H.); amanda.hudson@sydney.edu.au (A.H.); emily.colvin@sydney.edu.au (E.C.); hannah.kamitakahara@gmail.com (H.K.); Rozelle.harvie@sydney.edu.au (R.H.); Csilla.hasovits@sydney.edu.au (C.H.); viive.howell@sydney.edu.au (V.M.H.); 4 Mater Research Institute, Translational Research Institute, The University of Queensland, Woolloongabba, QLD 4102, Australia; tashbib.khan@uq.net.au (T.K.); yaowu.he@mater.uq.edu.au (Y.H.); john.hooper@mater.uq.edu.au (J.D.H.); 5 Centre for Data Science, Queensland University of Technology, Brisbane, QLD 4059, Australia; 6 Institute of Clinical Medicine, Faculty of Medicine, University of Oslo, 0136 Oslo, Norway; 7 Department of Medical Genetics, Oslo University Hospital, 0379 Oslo, Norway; 8 University of Queensland Diamantina Institute, Faculty of Medicine, The University of Queensland, Brisbane, QLD 4102, Australia; 9 Cancer Services, Princess Alexandra Hospital, Ipswich Road, Woolloongabba, QLD 4102, Australia

**Keywords:** non-small-cell lung cancer (NSCLC), epidermal growth factor receptor (EGFR), tyrosine kinase inhibitor (TKI), cell division cycle-associated protein 3 (CDCA3), acquired resistance, biomarker

## Abstract

**Simple Summary:**

Resistance to tyrosine kinase inhibitors (TKIs) that target common non-small-cell lung cancer mutations within the epidermal growth factor receptor (EGFR) is a primary clinical issue. The aim of our study was to determine whether the protein cell division cycle-associated protein 3 (CDCA3) might be a biomarker for TKI response in EGFR mutant lung cancer. Our previous work has demonstrated that CDCA3 is a marker of chemotherapy sensitivity in lung cancer. We provide evidence that CDCA3 levels are increased in EGFR mutant lung cancer and these levels are associated with sensitivity to TKIs. In addition, increasing the levels of CDCA3 enhances TKI sensitivity in models of TKI-resistant EGFR mutant lung cancer. Our findings propose that strategies to upregulate CDCA3 levels might improve TKI response in EGFR mutant lung cancer.

**Abstract:**

Tyrosine kinase inhibitors (TKIs) are the first-line therapy for non-small-cell lung cancers (NSCLC) that harbour sensitising mutations within the epidermal growth factor receptor (EGFR). However, resistance remains a key issue, with tumour relapse likely to occur. We have previously identified that cell division cycle-associated protein 3 (CDCA3) is elevated in adenocarcinoma (LUAD) and correlates with sensitivity to platinum-based chemotherapy. Herein, we explored whether CDCA3 levels were associated with EGFR mutant LUAD and TKI response. We demonstrate that in a small-cohort tissue microarray and in vitro LUAD cell line panel, CDCA3 protein levels are elevated in EGFR mutant NSCLC as a result of increased protein stability downstream of receptor tyrosine kinase signalling. Here, CDCA3 protein levels correlated with TKI potency, whereby CDCA3^high^ EGFR mutant NSCLC cells were most sensitive. Consistently, ectopic overexpression or inhibition of casein kinase 2 using CX-4945, which pharmacologically prevents CDCA3 degradation, upregulated CDCA3 levels and the response of T790M(+) H1975 cells and two models of acquired resistance to TKIs. Accordingly, it is possible that strategies to upregulate CDCA3 levels, particularly in CDCA3^low^ tumours or upon the emergence of therapy resistance, might improve the response to EGFR TKIs and benefit patients.

## 1. Introduction

Lung cancer is the leading cause of cancer-related mortality worldwide [1,2] with a poor 5 year survival rate of 19% [3]. Non-small-cell lung cancer (NSCLC) is the most common form of lung cancer and can be broadly subdivided into the squamous cell carcinoma (SqCC) and adenocarcinoma (LUAD) histologic subtypes [4]. Sensitising mutations within epidermal growth factor (EGF) receptor (*EGFR*) are a recognised driver of LUAD that predict response to tyrosine kinase inhibitors (TKI) [5,6]. Activating *EGFR* mutations constitute approximately 15% of all NSCLC cases [7]. EGFR mutations are predominantly located in the catalytic tyrosine kinase domain of EGFR as a small in-frame deletion within exon 19 (E746_A750del) or as a leucine to arginine point mutation at codon 858 (L858R) within exon 21 [7] yielding a constitutively activated receptor [8].

To target tumours with these activating *EGFR* mutations, competitive, reversible first-generation TKIs were developed: erlotinib and gefitinib. The first-generation TKIs are functional against the activating EGFR mutations of L858R and exon 19 del by inhibiting the autophosphorylation of the EGF receptor at the C-terminal tail and subsequently the activity of EGFR [9,10]. However, patients who initially respond to the first-generation TKIs eventually develop disease progression at approximately 9–14 months post-treatment [11,12,13]. Further investigation identified a threonine to methionine mutation at codon 790 (T790M) within exon 20 in tumours of patients who relapse following treatment with first-generation EGFR TKIs [12,14,15]. A 2018 study looking at the worldwide frequency of commonly detected EGFR mutations identified that the T790M mutation occurs in 0.7% of all NSCLC cases [16]. Furthermore, the T790M mutation accounts for approximately half of all cases with resistance to gefitinib and erlotinib [17,18]. Afatinib was introduced as a second-generation EGFR TKI. Third-generation TKIs, such as osimertinib, were subsequently developed to more selectively target acquired EGFR mutations [11]. Osimertinib, AZD9291, was first described as an EGFR TKI capable of selectively and irreversible targeting both sensitising and resistant T790M(+) mutant EGFR whilst harbouring less activity toward wild-type EGFR, a pitfall of the second-generation TKI afatinib [11]. Osimertinib is now the standard-of-care first-line therapy for advanced NSCLC harbouring sensitising *EGFR* mutations based on improved survival when compared with earlier-generation EGFR TKIs [19,20].

While these therapies prolong patient survival and improve patient quality of life, relapse is still common. In the case of osimertinib, additional mutations mediating resistance to the third-generation EGFR TKI have begun to emerge, bypassing the T790M mutation [15,21,22]. Ultimately, these activating *EGFR* mutations impact treatment outcomes by resisting TKI activity and promoting aberrant EGFR signalling.

The identification of novel biomarkers that are predictive of tumour relapse or TKI resistance is key to improving the health outcomes for NSCLC patients with tumours harbouring *EGFR* activating mutations. More recently, we have identified novel protein-based NSCLC biomarkers with prognostic potential [23] including cell division cycle-associated protein-3 (CDCA3) [24,25]. CDCA3, also referred to as trigger of mitosis entry 1 (TOME-1), was first reported as a modulator of cell cycle progression for entry into mitosis from the G2 phase [26]. CDCA3 was defined as an F-box-like protein that associated with and formed part of the SKP1-Cullin-RING-F-box containing (SCF) ubiquitin ligase (E3) protein complex. This complex degrades Wee1, a CDK1 inhibitory tyrosine kinase, via ubiquitination to move past the G2/M phase checkpoint and induce mitotic entry [27]. While other molecular functions for CDCA3 are yet to be determined, there are also emerging roles for CDCA3 in solid malignancies; upregulated CDCA3 expression has been noted in liver cancer, gastric cancer, colon cancer, oral squamous cell carcinoma, and breast cancer [28,29,30]. We previously identified that CDCA3 was elevated in NSCLC, and its expression was strongly prognostic and high expression was significantly associated with LUAD [24]. More recently, we have identified that elevated levels of CDCA3 also correlate with response to platinum-based chemotherapy, whereby CDCA3^high^ tumours exhibit greater sensitivity to therapy [25]. Our findings suggest that CDCA3 may be useful as a prognostic or predictive biomarker or potential therapeutic target in NSCLC.

In this present study, we examined the potential role of CDCA3 in EGFR mutant NSCLC resistance and whether modulation of CDCA3 levels within the resistant setting could impact EGFR TKIs sensitivity. Our data demonstrate that upregulated CDCA3 promotes enhanced TKI sensitivity across several models of TKI-resistant NSCLC.

## 2. Materials and Methods

### 2.1. Antibodies and Reagents

The following antibodies were purchased from Cell Signaling Technology (Genesearch, Arundel, Australia): phospho-ERK (#4370), total ERK (#4695), phospho-mTOR (#5536), total mTOR (#2983), phospho-Akt (#4060), total Akt (#4685), phospho-EGFR (#3777), total EGFR (#4267), phospho-Histone H3 (ser10, #53348) and phospho-CK2 substrate motif antibody (#8738). The α-Tubulin antibody (T9026) was purchased from Sigma Aldrich (Castle Hill, Australia) and actin antibody (612656) was from BD Transduction Laboratories (North Ryde, Australia). The CDCA3 antibody (HPA026587, Sigma Aldrich) was used for immunohistochemistry. The sheep anti-CDCA3 antibody was used for Western blot analysis and generated by the MRC Protein Phosphorylation and Ubiquitylation Unit at the University of Dundee and validated in CDCA3-depleted cells (see Figure S1) and ectopic CDCA3-expressing cells. All secondary antibodies were purchased from Life Technologies. 4′-6-diamidino-2-phenylindole (DAPI) was from Life Technologies, Complete EDTA-free protease inhibitor was from Roche Applied Sciences (Castle Hill, Australia) and phosphatase inhibitor cocktail (#5870) was from Cell Signal Technology. CX-4945, erlotinib and osimertinib were purchased from Selleck Chemicals Llc (Sapphire Bioscience, Redfern, Australia). All other reagents were purchased from Sigma Aldrich.

### 2.2. Cell Culture, Transfections and Cell Treatments

All NSCLC cell lines were obtained from the American Type Culture Collection (ATCC) except for PC-9 cell line which was sourced from the European Collection of Cell Cultures (ECACC). Cells were grown in RPMI-1640-medium containing L-glutamine (Life Technologies, Mulgrave, Australia) and 10% foetal bovine serum (FBS, Sigma Aldrich). HCC827 and PC-9 parental and erlotinib-resistant cells generated by cyclic stepwise exposure to escalating doses of erlotinib. Isogenic parental cell lines not exposed to erlotinib were maintained in culture over the same period. The A549, H460 and H1299 cell lines are EGFR wild-type, whereas the H1650, HCC827, PC-9 cell lines have EGFR exon 19 deletions (E746_A750del), while H3255 cells are exon 21 EGFR mutant (L858R) and H1975 cells harbour both the L858R mutation and the T790M gatekeeper *EGFR* mutation. All cell lines were cultured at 37 °C in a humidified 5% CO_2_ incubator and routinely tested for mycoplasma contamination.

Lipofectamine RNAimax (Life Technologies) was used to transfect previously validated [24] negative control Stealth siRNAs and Stealth siRNAs (siCDCA3-1, sense 5′-ACUGGUGAAACAGCUGAGUGAAGUA-3′, anti-sense 5′-UACUUCACUCAGCUGUUUCACCAGU-3′) to deplete CDCA3 levels (ThermoFisher Scientific). Transfection of CDCA3-FLAG expression construct [25] was performed using the FuGene HD transfection reagent (Promega Corporation, Annandale, Australia).

For cell treatments prior to Western blot analysis, serum-starved cells (18 h) were treated with EGF (20 ng/mL) over 24 h. Cells maintained in serum were treated with CX-4945 (5 µM), erlotinib (0.1 µM for H1975 cells) or osimertinib (50 nM) for 24 h. For protein half-life experiments, HCC827 cells were exposed to erlotinib (50 nM) for 1 h before treatment with cycloheximide (70 µM) over 6 h in the presence or absence of erlotinib.

### 2.3. Immunohistochemistry

The Ethics Committee approved tissue microarray of EGFR wild-type and mutant NSCLC tissue was purchased from Tristar Technology Group (cat. No. 69572826-2826) and handled in accordance with the guidelines and regulations approved by Queensland University of Technology (approval number 1900000269). Immunohistochemistry was performed as previously described using the validated CDCA3 antibody [24]. Briefly, slides were subsequently deparaffinised, rehydrated, washed, and quenched. The slides were incubated in Tris/EDTA to perform antigen retrieval before they were washed with 1 X PBS and blocked with blocking solution. Staining for CDCA3 was performed by incubating the slides in CDCA3 primary antibody diluted (1:100) in Da Vinci Green (Biocare Medical (MetaGene, Redcliffe, Australia)) with overnight incubation. The slides were washed and stained with an appropriate secondary antibody using Universal MACH2 HRP polymer detection kit (Biocare Medical). The slides were then dehydrated, cleared, and mounted. Staining was evaluated and semi-quantitatively evaluated using the H-score which incorporated scores for intensity (scale from 0–3) and the percentage of tumour stained for CDCA3 (in 10% increments). Levels of CDCA3 were dichotomised according to above or below the median score (80) as CDCA3^high^ or CDCA3^low^, respectively.

### 2.4. Lysate Collection and Western Blot Analyses

To collect whole cell lysates, cells were first washed in PBS then lysed in lysis buffer (50 mM HEPES (pH 7.5), 150 mM KCl, 5 mM EDTA, 0.05% IGEPAL CA-630 (*v/v*), 1 × protease inhibitor cocktail (Roche), and 1 × phosphatase inhibitor cocktail (Cell Signalling Technology). Total protein was determined by bicinchoninic acid (BCA) protein assay (Sigma Aldrich) following lysate sonication and centrifugation. Samples (total protein 20 µg) were denatured in 1 × Laemmli Buffer supplemented with 8% β-mercaptoethanol for 5 min at 80 °C.

The samples were separated on Bolt 4–12% Bis-Tris Plus pre-cast gels (Invitrogen) and transferred onto nitrocellulose membrane (GE Healthcare Life Sciences, Springfield Central, Australia) using the semi-dry Novex Xcell II Blot Module transfer system (Life Technologies). The nitrocellulose membranes were blocked using Odyssey blocking buffer (Li-Cor) before incubation overnight at 4 °C with primary antibody in a 1:1 solution of PBS-T and Odyssey Blocking Buffer. Following primary antibody incubation, membranes were washed with PBS-T and incubated with the appropriate secondary antibodies. Membranes were scanned and imaged using the Li-Cor Odyssey (Li-Cor, Millennium Science, Mulgrave, Australia). Images were acquired and subject to densitometry analysis using the Image Studio Lite software.

### 2.5. Immunofluorescence, High-Content Microscopy and Analysis

High-content immunofluorescence and imaging for mitotic index were performed as previously described [25]. Briefly, cells seeded in glass bottom 96-well plates were fixed with 4% paraformaldehyde for 20 min at ambient temperature and permeabilized with 0.1% Trion X-100 in PBS for 5 min. Cells were blocked with 2% donkey serum in PBS before incubation with an anti-Histone H3 (S10) antibody overnight at 4 °C, used at a dilution of 1:1000. Alexa Fluor^®^ secondary antibodies were incubated for 1 h at ambient temperature in 0.5% donkey serum in PBS before DAPI staining. Images were collected using an InCell Analyser 6500 high-content microscopy system (GE Healthcare Life Sciences). Mitotic index was calculated from images using the CellProfiler software v3.1.9 and reported as percentage of cells positive for S10 Histone H3 staining per field of view from a minimum of 1000 cells.

### 2.6. Cell Viability Assays

Cells were seeded into a white-walled, glass-bottomed 384-well plate (Nunc) at a density of 1 × 103 cells per well. The cells were treated with escalating doses of erlotinib, osimertinib or CX-4945 24 h following seeding over a period of 72 h. Cell viability was determined using CellTitre-Glo 2.0 (Promega Corporation) according to the manufacturer’s instructions. Luminescence was scanned and analysed on the FLUOstar Omega Microplate Reader (BMG Labtech, Mornington, Australia). Data were normalised to untreated controls and dose–response curves and drug potency values generated using GraphPad Prism V9 software.

### 2.7. Cell Proliferation

Cells were seeded into a clear-walled, plastic-bottomed 96-well plate (Nunc) at a cell density of 1 × 10^3^ cells per well. At least one region of each well was imaged every 2 h over 96 h using the Incucyte Zoom or Incucyte S3 (Essen BioScience, Ann Arbor, MI, USA). Cell proliferation image analysis was conducted using the Incucyte Zoom software package. Data were normalised to untreated controls and proliferation curves were generated using GraphPad Prism V9 software.

### 2.8. Bioinformatics Analysis

CDCA3 mRNA expression levels were determined from TCGA RNA-seq datasets of EGFR wild-type LUAD NSCLC and EGFR mutant LUAD NSCLC. CDCA3 expression levels were correlated against the publicly available WikiPathway “EGFR tyrosine kinase inhibitor resistance” parameter [31] by linear regression analysis with P and R values calculated according to Spearman’s rank correlation. Analyses were performed in the R statistical environment (R Core Team, Vienna, Austria). Cell line CDCA3 expression levels were determined from RNA-seq data accessed through cBioPortal [32].

### 2.9. Statistical Analysis and Reproducibility

Statistical analysis was conducted using GraphPad Prism V9 software. Results are displayed as mean ± SD from at least 3 independent experiments. Statistical significance was determined using the chi-squared or two-tailed Student’s t tests. *P* values below 0.05 were considered significant and denoted as * *p* ≤ 0.05 and ** *p* ≤ 0.005.

## 3. Results

### 3.1. CDCA3 Protein Is Upregulated and Stabilised in EGFR Mutant NSCLC

**Figure 1:**
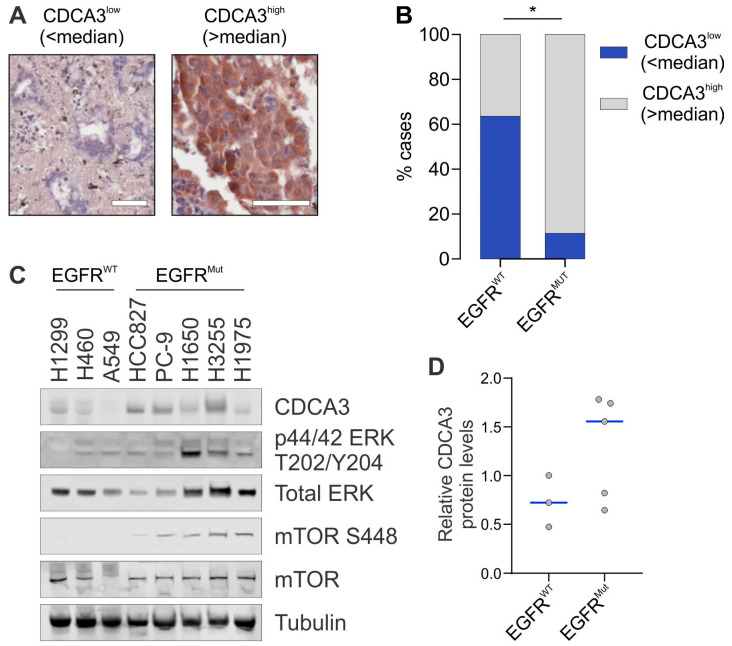
CDCA3 protein is upregulated in EGFR mutant NSCLC. (**A**) Representative images of CDCA3^low^ (left panel) and CDCA3^high^ (right panel) staining by immunohistochemistry in NSCLC cohort. CDCA3 levels determined by stratifying on median H-score. Scale bar = 50 µm. (**B**) Stacked bar chart showing distribution of CDCA3^low^ (blue) and CDCA3^high^ (grey) levels quantified from immunohistochemistry staining of non-oncogene-driven NSCLC (EGFR-WT) cases and EGFR mutant cases (chi-square test, * *p* = 0.02). (**C**) Representative endogenous CDCA3 Western blot analysis from lysates of NSCLC adenocarcinoma cell lines evaluating a small panel of non-oncogene-driven (EGFR WT) and EGFR mutant (EGFR MUT) cell lines. Phosphorylated and total ERK and mTOR Western blot analysis to assess constitutive signalling in EGFR mutant cell lines. Tubulin used as a loading control. (**D**) Densitometry quantification of (**C**), with dot points representing relative CDCA3 levels from three independent experiments. Blue lines indicate median values.

To determine the expression of CDCA3 in non-oncogene-driven EGFR wild-type LUAD NSCLC and EGFR mutant NSCLC, we examined protein levels by immunohistochemistry on a small-cohort tissue microarray of 27 cases containing 8 EGFR mutant cases. Within the *EGFR* mutant cases, 4 cases contained exon 19 del with the remaining cases containing the exon 21 mutation L858R. Of the 26 evaluable cases, CDCA3 staining was scored by the H-score method with low or high expression level determined by dichotomising cases based upon median value with representative staining shown in Figure 1A. Elevated CDCA3 staining was observed in 37% of EGFR wild-type cases whereas ~88% of EGFR mutant cases displayed elevated CDCA3 staining (Figure 1B). As shown in Figure 1B, high expression of CDCA3 was significantly associated with EGFR mutant NSCLC (*p* = 0.02).

Given the association in NSCLC tumours, we next examined CDCA3 expression in a small panel of LUAD cell lines. Western blot analysis was performed evaluating CDCA3 and constitutive activation of MAPK (phospho-ERK) and PI3K-Akt-mTOR (phospho-mTOR) downstream signalling across three EGFR wild-type (H1299, H460, A549) and five EGFR mutant (HCC827, PC-9, H1650, H3255, H1975 (see Materials and Methods for specific mutations)) cell lines. These data revealed that, overall, median CDCA3 protein was ~1.8-fold higher in EGFR mutant cell lines than in EGFR wild-type cell lines (Figure 1C,D). Two cell lines (H1650 and H1975) exhibited CDCA3 protein levels below the median expression. Consistent with other studies [33,34,35], constitutive phosphorylation of ERK and mTOR was observed in each EGFR mutant cell line (Figure 1C).

**Figure 2:**
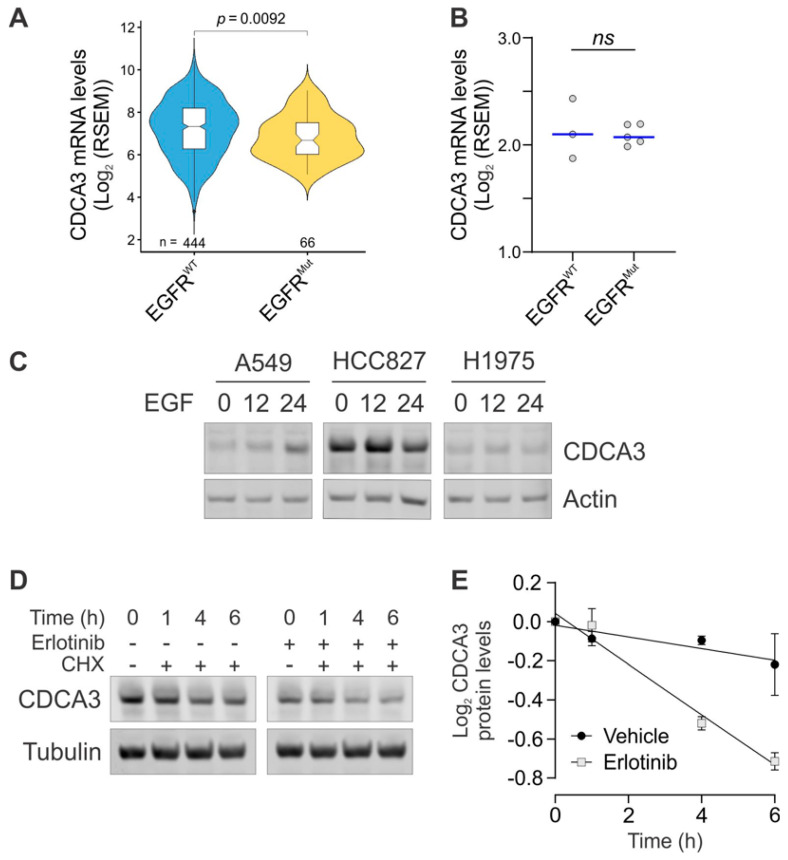
Upregulation of CDCA3 protein levels in EGFR mutant NSCLC is dependent on RTK signalling. (**A**) Violin plots showing relative *CDCA3* mRNA levels in EGFR wild-type and EGFR mutant adenocarcinoma from The Cancer Genome Atlas (TCGA) RNAseq datasets. (**B**) RNAseq analysis of *CDCA3* mRNA levels in cell lines examined in Figure 1C comparing EGFR wild-type and EGFR mutant adenocarcinoma. ns, not significant. (**C**) Endogenous CDCA3 Western blot analysis of A549 (EGFR WT), HCC827 (EGFR exon 19 deletion) and H1975 (EGFR T790M) cells treated in the absence or presence of EGF over 24 h. Phosphorylated (S473) and total Akt Western blot analysis to assess EGF-induced signal transduction. Actin used as a loading control. (**D**) Endogenous CDCA3 Western blot analysis of HCC827 lysates assessing protein turnover by treating with cycloheximide over 6 h in the presence or absence of erlotinib. Tubulin used as a loading control. (**E**) Densitometry quantification of (**D**), showing average log_2_ of relative CDCA3 protein levels relative to 0 h from three independent experiments.

We next assessed *CDCA3* transcript levels by bioinformatics analysis of TCGA datasets of LUAD NSCLC comparing EGFR wild-type and mutant tumours. As shown in Figure 2A, although statistically significant, *CDCA3* expression was marginally reduced in EGFR mutant LUAD compared with EGFR wild-type LUAD. To further analyse this, *CDCA3* transcripts were also assessed in publicly available RNA-seq analysis of NSCLC cell lines. As shown in Figure 2B, analysis of the same panel of NSCLC cell lines that were assessed in Figure 1C, indicated there to be no significant difference in *CDCA3* transcript expression between EGFR wild-type and mutant cell lines. These data are largely consistent with our microarray analysis which suggested that, in contrast to the protein levels, *CDCA3* transcript levels were similar between EGFR wild-type and mutant tumours [24].

As CDCA3 is predominantly increased at the protein level and not at the transcript level in EGFR mutant NSCLC, we next sought to determine if receptor tyrosine kinase activity might influence CDCA3 protein levels. To do so, we treated serum-starved A549 (EGFR wild-type) and HCC827 and H1975 cells (EGFR mutant) with the EGFR ligand EGF for 24 h. Western blot analysis revealed that EGF stimulation induced Akt phosphorylation (S473) and upregulated CDCA3 protein levels, maximally at 12 h in A549 cells (Figure 2C). EGF stimulation did not affect CDCA3 levels in EGFR mutant HCC827 or H1975 cells where Akt was constitutively phosphorylated. We next asked whether CDCA3 protein stability might be affected downstream of EGFR signalling, which might contribute to elevated protein levels. For protein half-life experiments, we used EGFR mutant HCC827 cells (exon 19 del) which have detectable levels of endogenous CDCA3 and are sensitive to the first-generation TKI erlotinib. HCC827 cells were treated with cycloheximide to prevent protein synthesis together with or without erlotinib over 6 h. As shown in Figure 2D, Western blot analysis revealed that endogenous CDCA3 levels were reduced by ~15% in untreated cells. In contrast, erlotinib treatment markedly enhanced CDCA3 turnover yielding a ~40% reduction in protein levels, suggesting that this protein is degraded following EGFR inhibition.

Collectively, these data highlight that CDCA3 protein is elevated in the majority of EGFR mutant LUAD, whereby signalling downstream of EGFR might contribute to sustaining CDCA3 protein levels.

### 3.2. CDCA3 Correlates with Sensitivity to EGFR TKIs

**Figure 3:**
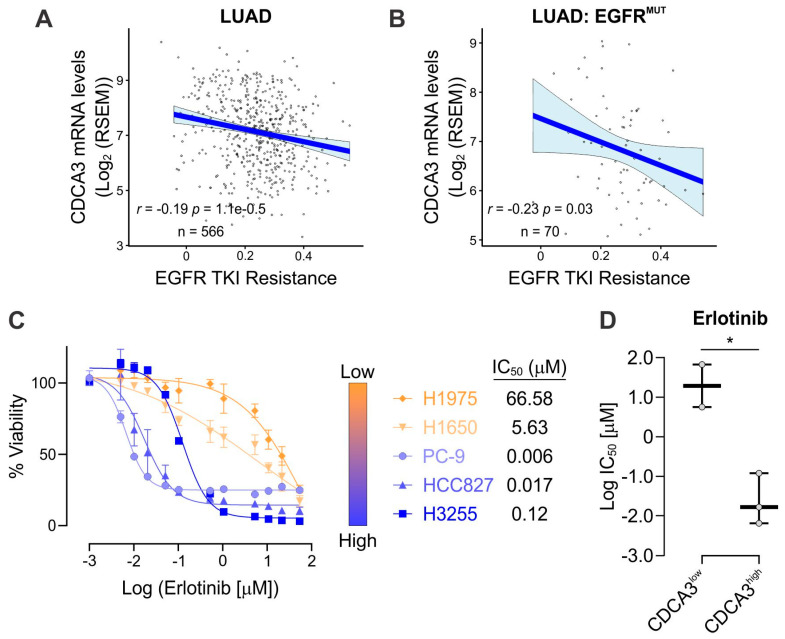
CDCA3 correlates with sensitivity to EGFR TKI. (**A**,**B**) Scatter plots showing linear regression analysis of The Cancer Genome Atlas (TCGA) RNAseq datasets assessing the correlation between *CDCA3* levels and WikiPathways of TKI resistance in (**A**) adenocarcinoma (LUAD) and (**B**) EGFR mutant LUAD. *R* and *p* values determined according to Spearman’s rank correlation. (**C**) Left panel, Dose–response curves for five EGFR mutant NSCLC cell lines treated with escalating doses of erlotinib. Cells were treated with erlotinib for 72 h before assessing cell viability. Right panel, Cell lines ranked by CDCA3 protein levels where erlotinib potency values (IC_50_) were calculated using GraphPad Prism and listed for each cell line. *n* = 4. (**D**) Box and whisker plot showing erlotinib potency (log IC_50_ value) in CDCA3^low^ and CDCA3^high^ EGFR mutant cell lines (unpaired Student’s *t* test, * *p* = 0.0187).

Given we have previously demonstrated that CDCA3 strongly correlates with platinum-based chemotherapy response in all NSCLC histologies [25], we next sought to determine whether a similar trend might exist with the response to EGFR TKIs. We evaluated TCGA datasets and correlated relative *CDCA3* expression against available WikiPathways [31] of known measures of TKI resistance. In LUAD (Figure 3A) and specifically in EGFR mutant LUAD (Figure 3B), *CDCA3* expression negatively correlated with EGFR TKI resistance with respective Spearman correlation coefficients of *R* = −0.19 and *R* = −0.23. To experimentally confirm these clinical data correlations, we sought to investigate correlations between CDCA3 expression and in vitro TKI potency (IC_50_) in a small panel of EGFR mutant NSCLC cell lines. Both erlotinib (Figure 3C) and osimertinib (Figure S1A) reduced cell viability in a dose-dependent manner. Consistent with other studies [36,37], H1975 cells, which harbour the T790M gatekeeper *EGFR* mutation, demonstrated resistance to erlotinib (IC_50_ = 66.58 µM) yet sensitivity to osimertinib (IC_50_ = 51 nM). Relative to other cell lines, H1650 cells were insensitive to both erlotinib and osimertinib. We correlated relative CDCA3 protein levels, as determined in Figure 1C by Western blot analysis, with the TKI potency values. Consistent with our bioinformatics analyses, CDCA3^high^ cell lines demonstrated greatest sensitivity to erlotinib (*p* = 0.019, Figure 3D) and although not statistically significant, CDCA3^high^ cell lines also exhibited greatest sensitivity to osimertinib (Figure S1B).

### 3.3. Upregulating CDCA3 Protein Levels Enhances TKI Sensitivity in CDCA3^low^ H1975 Cells

**Figure 4:**
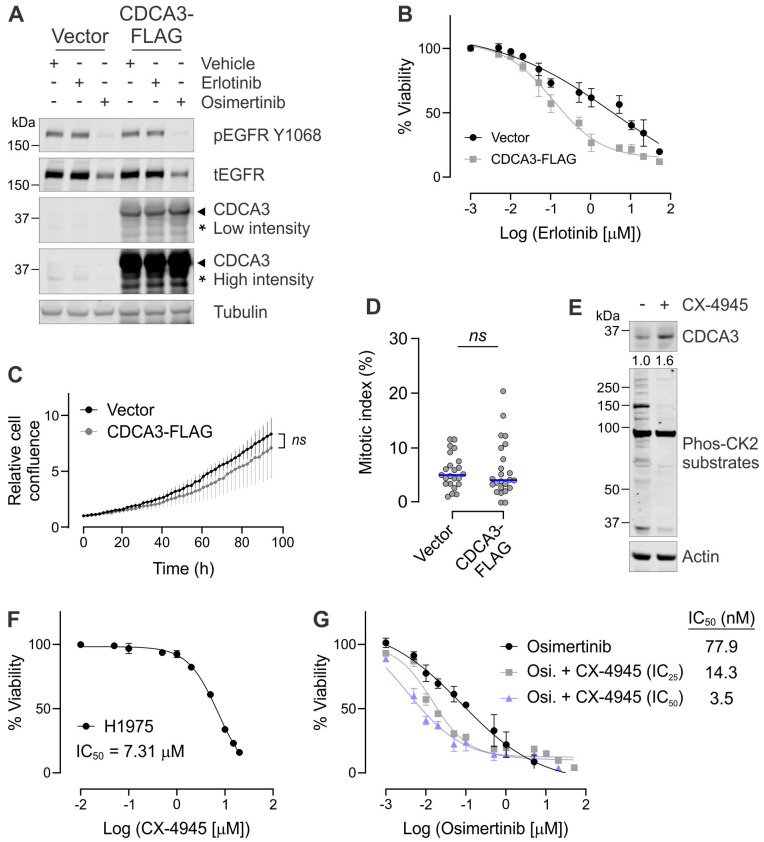
Upregulating CDCA3 protein levels enhances TKI sensitivity in CDCA3^low^ H1975 cells. (**A**) Western blot analysis of lysates from H1975 cells transfected with empty vector or CDCA3-FLAG treated in the absence (vehicle) or presence of erlotinib or osimertinib for 24 h. CDCA3 Western blot analysis was performed to detect ectopic (arrowhead) and endogenous (asterisk) CDCA3. Phosphorylated (Y1068) and total EGFR Western blot analysis was performed to confirm TKI response. Tubulin was used as a loading control. Representative Western blot analysis from three independent experiments. (**B**) Dose–response curves for CDCA3^low^ H1975 cells either vector transfected or ectopically overexpressing CDCA3-FLAG treated with escalating doses of erlotinib for 72 h before assessing cell viability. (**C**) Cellular proliferation analysis of vector transfected or CDCA3-FLAG ectopically expressing H1975 cells over 96 h using the Incucyte Zoom live-cell imaging system. (**D**) Beeswarm plot showing the mitotic index determined by histone H3 pS10 staining and high throughput immunofluorescence microscopy of vector transfected or CDCA3-FLAG ectopically expressing H1975 cells. Data points represent an average percentage of mitotic nuclei per field of view from a minimum of 1100 nuclei (*n* = 23 fields total). Blue lines indicate median values. (**E**) Endogenous CDCA3 Western blot analysis of H1975 cells treated in the absence or presence of CX-4945 (5 µM) over 24 h. Phosphorylated CK2 substrate probe to assess impact of CK2 inhibition with CX-4945. Tubulin was used as a loading control. (**F**) Dose–response curve for H1975 cells treated with escalating doses of CX-4945. Cells were treated with CX-4945 for 72 h before assessing cell viability. CX-4945 potency values (IC_50_) were calculated using GraphPad Prism and listed for each cell line. *n* = 3. (**G**) Dose–response curves showing the impact of combining IC_25_ or IC_50_ CX-4945 concentrations with osimertinib in H1975 cell. Potency values (IC_50_) were calculated using GraphPad Prism. *n* = 3.

As our bioinformatics and in vitro analyses suggest that CDCA3^high^ EGFR mutant LUAD is more responsive to EGFR TKI than CDCA3^low^ EGFR mutant LUAD, we sought to determine whether modulating CDCA3 levels might affect TKI potency. Depletion of CDCA3 did not impact erlotinib resistance in H1975 cells (Figure S1C) or the sensitivity of CDCA3^high^ PC-9 cells to osimertinib (Figure S1D). We next ectopically expressed CDCA3 in H1975 which are endogenously CDCA3^low^. Ectopic expression of CDCA3 in H1975 did not impact the phosphorylation status of EGFR in response to first- or third-generation TKI (Figure 4A). However, increasing CDCA3 levels markedly enhanced the potency of erlotinib in H1975 cells which are otherwise resistant, reducing the IC_50_ value by ~33-fold (Figure 4B). As CDCA3 is suggested to modulate G2-M cell cycle progression [24,26,27], we determined that ectopic expression of this protein did not significantly impact cellular proliferation (Figure 4C) or the mitotic index of H1975 cells (Figure 4D). These data suggest that ectopic expression of CDCA3 enhances TKI sensitivity possibly independent of its known roles in cell cycle modulation.

To explore strategies to upregulate CDCA3 and improve TKI response, we evaluated the utility of the casein kinase-2 (CK2) inhibitor CX-4945 in H1975 cells. We have previously identified that CDCA3 degradation, by the ubiquitin ligase anaphase-promoting complex/cyclosome (APC/C) and cofactor Cdh1, is phosphorylation-dependent, whereby CK2 blockade with CX-4945 prevents CDCA3 degradation [25]. Western blot analysis of H1975 revealed that 24 h treatment with CX-4945 induced a 1.6-fold increase in endogenous CDCA3 protein and a consequent decrease in total CK2 substrate immunoreactivity (Figure 4E). We next sought to investigate the possibility of combining IC_25_ and IC_50_ concentrations of CX-4945 with TKI in CDCA3^low^ H1975 cells. CX-4945 alone reduced H1975 cell viability in a dose-dependent manner with an IC_50_ value of 7.31 µM (Figure 4F). As shown in Figure 4G, combining osimertinib with IC_25_ and IC_50_ concentrations of CX-4945 markedly enhanced osimertinib potency by ~5 and ~22-fold, respectively.

### 3.4. Upregulating CDCA3 Protein Levels in Models of Acquired EGFR TKI Resistance Enhances TKI Sensitivity

**Figure 5:**
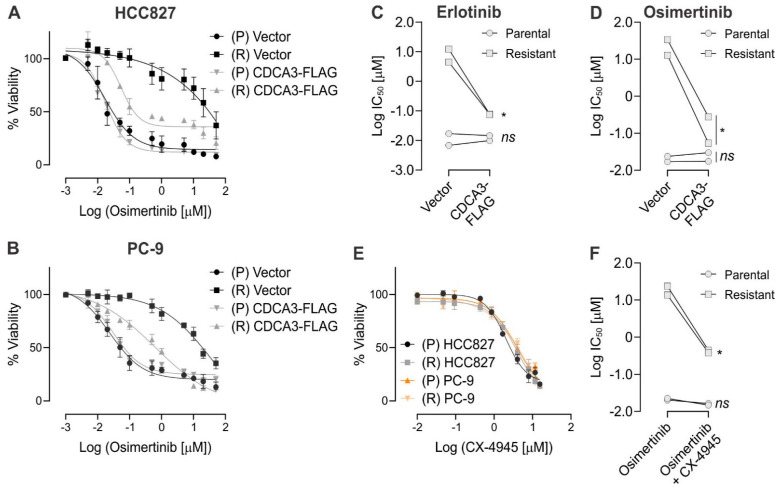
Upregulating CDCA3 protein levels in models of isogenic EGFR TKI resistance cells enhances TKI sensitivity. (**A**,**B**) Dose–response curves for vector transfected or ectopic CDCA3-FLAG overexpression in isogenic parental and resistant (**A**) HCC827 and (**B**) PC-9 cells treated with escalating doses of osimertinib for 72 h before assessing cell viability. (**C**) Plot showing erlotinib potency (log IC_50_ value) comparing vector transfected and ectopic CDCA3-FLAG overexpression in isogenic parental and TKI-resistant cell lines determined in Figure S2 from four independent experiments. Lines connect respective isogenic parental and resistant cell lines (2-way ANOVA, * *p* = 0.029, ns = not significant). (**D**) Plot showing osimertinib potency (log IC_50_ value) comparing vector transfected and ectopic CDCA3-FLAG overexpression in isogenic parental and TKI-resistant cell lines determined in (**A**,**B**) from four independent experiments with lines connecting respective isogenic parental and resistant cell lines (2-way ANOVA, * *p* = 0.0339, ns = not significant). (**E**) Dose–response curves for HCC827 and PC-9 isogenic parental and TKI-resistant cell lines treated with escalating doses of CX-4945. Cells were treated with CX-4945 for 72 h before assessing cell viability. CX-4945 potency values (IC_50_) were calculated using GraphPad Prism and listed for each cell line. *n* = 3. (**F**) Plot showing potency (log IC_50_ value) of osimertinib alone or in combination with CX-4945 (IC_50_) comparing isogenic parental and TKI-resistant cell lines from three independent experiments. Lines connect respective isogenic parental and resistant cell lines (2-way ANOVA, * *p* = 0.0331, ns = not significant).

Our findings suggest strategies that upregulate CDCA3 might prove useful to enhance the sensitivity of EGFR mutant NSCLC to TKIs. We sought to assess this possibility in in vitro models of acquired TKI resistance. To do so, we generated TKI-resistant models in HCC827 and PC-9 cell lines by exposing cells to cycles of erlotinib. Cells not exposed to TKI were also maintained in culture for the same period, hereafter referred to as the parental cells. As shown in Figure S2A,B, resistant HCC827 and PC-9 demonstrated marked erlotinib resistance with respective IC_50_ values of 6.19 µM and 4.45 µM compared with IC_50_ values in the isogenic parental cells of 0.017 µM and 0.006 µM, respectively. Although resistance was generated using erlotinib, these models also exhibited marked osimertinib resistance with ~385-fold and ~1470-fold increase in IC_50_ values versus parental HCC827 (Figure 5A) and PC-9 cell lines, respectively (Figure 5B). Although the precise mechanism of TKI resistance remains to be determined in these models, we were unable to detect the emergence of T790M gatekeeper mutations in either cell line model by whole exome sequencing (data not shown).

Having established two in vitro TKI acquired resistance models of EGFR mutant NSCLC, we next ectopically expressed CDCA3 in the parental and resistant pairs of each cell line. Ectopic expression of CDCA3 did not impact the sensitivity of HCC827 or PC-9 parental cells to erlotinib (Figure S2A,B and Figure 5C) or osimertinib (Figure 5A,B,D). In contrast, ectopic CDCA3 expression significantly enhanced the potency of erlotinib (Figure S2A,B and Figure 5C) and osimertinib (Figure 5A,B,D) by ~79 and ~121-fold, respectively in HCC827-resistant cells. Similarly, ectopic CDCA3 expression significantly enhanced erlotinib and osimertinib potency by ~57 and 54-fold, respectively in PC-9-resistant cells. To assess whether these observations were not due to impacts on cell cycle progression, we confirmed that ectopic CDCA3 expression did not significantly impact the mitotic index of isogenic HCC827 parental or TKI-resistant cells (Figure S2C) or cellular proliferation (Figure S2D). Of note, no significant difference in mitotic index or overall proliferation rate over 96 h was observed between the isogenic parental and resistant cells.

We next investigated the potential for upregulating CDCA3 levels via CK2 inhibition in both isogenic models of TKI resistance. As shown in Figure 5E, CX-4945 induced a consistent dose-dependent decrease in cellular viability across both models of parental and TKI-resistant cell lines with IC_50_ values ranging between 4.76 µM and 8.33 µM. Combining osimertinib with the respective CX-4945 IC_50_ values for each parental or resistant cell line was next evaluated. As shown in Figure 5F, CX-4945 did not impact the potency of osimertinib in either of the parental cells. However, consistent with ectopic CDCA3 expression, CX-4945 markedly enhanced the potency of osimertinib in both isogenic-resistant cell lines (*p* = 0.0057). Together, these data further suggest that strategies to increase CDCA3 levels might improve TKI potency in therapy-resistant disease.

## 4. Discussion

Identifying resistance mechanisms to EGFR TKIs together with implementing predictive biomarkers is essential to improve the management of EGFR mutant NSCLC. Although osimertinib, as a third-generation and irreversible TKI, is now the front-line treatment for EGFR mutant NSCLC with superior central nervous system and survival outcomes [19,20], tumour relapse is inevitable. Current osimerinib and TKI biomarker discovery efforts range from non-invasive imaging and liquid biopsy [38,39,40] to genetic and protein-based tissue biopsy screening [41,42,43]. In the current study, our findings point to the possibility of exploiting expression of CDCA3 as a strategy to identify TKI responsive EGFR mutant tumours. We identified that levels of CDCA3 protein were largely elevated downstream of activated RTKs in EGFR mutant NSCLC cells (Figure 1 and Figure 2). In tumours and EGFR mutant cell lines, we further identified that CDCA3 correlates with sensitivity to TKI (Figure S1 and Figure 3).

A key finding from this study is that strategies to increase CDCA3 protein levels might enhance TKI sensitivity. We document that ectopic overexpression of CDCA3 markedly enhanced sensitivity of the T790M(+) H1975 cell line to erlotinib (Figure 4) and that of two isogenic models of acquired TKI resistance to erlotinib and osimertinib (Figure 5). How upregulating CDCA3 levels might enhance TKI sensitivity remains to be determined. Importantly, our data suggest that these findings are independent of cell cycle control. As a reported modulator of mitotic entry, ectopic CDCA3 expression did not impact cellular proliferation or the mitotic index of three in vitro models of TKI-resistant EGFR mutant NSCLC (Figure 4 and Figure S2). It is possible that CDCA3 has distinct roles across multiple cellular mechanisms, perhaps even functioning uniquely in genetic or acquired therapy resistance given ectopic expression did not impact TKI sensitivity in isogenic parental HCC827 or PC-9 cells (Figure 5). Indeed, aurora kinase A (AURKA) is another recognised mitotic regulator that drives TKI resistance by functioning in multiple cellular pathways [44]. Pharmacological inhibition of AURKA, whose activity is elevated upon acquired resistance, synergised with TKIs to suppress resistant EGFR mutant NSCLC in vitro and in vivo. Further analysis is warranted to elucidate the molecular roles for CDCA3 in therapy-resistant NSCLC.

Our study also highlights that exploiting control of endogenous CDCA3 protein levels might enhance TKI sensitivity. Here, endogenously CDCA3^high^ EGFR mutant cell lines demonstrated greater TKI sensitive than CDCA3^low^ cell lines (Figure 3). Of note, although we assessed a small tissue microarray cohort and cell line panel, there does not appear to be an association between CDCA3 expression and specific *EGFR* mutations. For example, the CDCA3^high^ HCC827 cells and CDCA3^low^ H1650 cells both harbour exon 19 deletion mutations within *EGFR* yet have a ~300-fold difference in TKI potency (Figure S1 and Figure 3). Nevertheless, in line with our previous findings in EGFR wild-type NSCLC [25], endogenous CDCA3 protein levels appear regulated in CK2-dependent manner, at least in TKI-resistant cells (Figure 4 and Figure 5). Indeed, CK2 activity is reported in EGFR mutant NSCLC and linked with TKI resistance [45]. Furthermore, CK2 inhibition with CX-4945 has proven synergistic with chemotherapy or erlotinib in non-oncogene-driven and EGFR mutant NSCLC, respectively, by attenuating the PI3K-Akt pathway [46,47]. While it is clear that numerous CK2 substrates function in TKI-resistant NSCLC, our findings suggest that CDCA3, as a CK2 substrate, also contributes to modulating the TKI response and indeed the response to CX-4945. Further investigation is necessary to identify the precise role and function for CDCA3 downstream of CK2 in TKI-resistant NSCLC. Nonetheless, we demonstrate that CK2 inhibition upregulated CDCA3 levels in endogenously low H1975 cells and markedly enhanced osimertinib potency (Figure 4). To the best of our knowledge, our study is the first to demonstrate the utility of combining CX-4945 and osimertinib as a third-generation EGFR TKI, especially across several models of genetic and acquired TKI resistance.

It is worth noting that while our study points to a role for CK2 in the regulation of CDCA3 protein, our findings also suggest that active RTK and EGFR promote CDCA3 protein stability (Figure 2). Indeed, there is crossover between CK2 and EGFR signalling pathways [46,48] where CDCA3 is a possible substrate, likely in those CDCA3^low^ cells (Figure 4, [25]). Whether in certain settings, EGFR signalling counteracts CK2-mediated CDCA3 phosphorylation or negatively regulates phosphatase activity, as is reported for PP2A [49], to regulate CDCA3 protein levels remains to be determined. However, it is not likely that CDCA3 participates in RTK feedback loops as ectopic expression did not affect the EGFR phosphorylation status (Figure 4). As such, our findings further point to the possibility of unique roles for CDCA3 downstream of RTK activation.

Interestingly, in contrast to our findings, upregulation of CDCA3 in other tumour types is reported to contribute to therapy resistance. For example, in breast cancer cell lines and renal cell carcinoma, upregulated CDCA3 levels was associated with doxorubicin [50] and sunitinib [51] resistance, respectively. In these studies, CDCA3 levels were identified to be transcriptionally regulated via long or circular RNAs. In our hands, siRNA-mediated depletion of CDCA3, even in endogenously CDCA3^high^ cell lines, did not impact TKI potency (Figure S1). Whether there may be tumour type-specific roles or differences in mechanisms regulating CDCA3 transcription or protein levels between tumour types is not yet clear. While our study has not directly examined CDCA3 in the emergence TKI resistance, further clinical analysis is warranted, particularly to evaluate CDCA3 expression in matched cohorts of diagnostic and relapsed EGFR mutant NSCLC.

## 5. Conclusions

Our study highlights that upregulated CDCA3 expression across several models of TKI-resistant NSCLC, including genetic and two models of acquired resistance (Figure 5), promotes enhanced TKI sensitivity. Whilst our findings have uncovered a potential role for CDCA3 in TKI resistance, further work is required to define the underlying molecular mechanisms and function for this protein in EGFR mutant NSCLC. Our results support further investigation and analysis of larger cohorts to determine and validate the biomarker potential for CDCA3 in EGFR mutant NSCLC.

## Data Availability

The data that support the findings of this study are available from the corresponding author M.N.A. upon reasonable request.

## Supplementary Materials

The following are available online at https://www.mdpi.com/article/10.3390/cancers13184651/s1, Figure S1: CDCA3 depletion does not impact TKI sensitivity. Figure S2: Upregulating CDCA3 enhances TKI sensitivity in isogenic models of TKI resistance without impacting cell proliferation.
