# Supplementary material for: Elevating CDCA3 Levels Enhances Tyrosine Kinase Inhibitor Sensitivity in TKI-Resistant EGFR Mutant Non-Small-Cell Lung Cancer
Source: Cancers (Basel). 2021 Sep 16;13(18):4651. doi: 10.3390/cancers13184651 (PMC8466783; doi:10.3390/cancers13184651)
Supplement: Supplementary file 1 [file cancers-13-04651-s001.zip › cancers-1319327-supplementary.pdf]

Article

Supplementary Material: Elevating CDCA3 Levels Enhances Tyrosine Kinase Inhibitor Sensitivity in TKI-Resistant EGFR Mutant Non-Small-Cell Lung Cancer

Katherine B. Sahin [1], Esha T. Shah [1], Genevieve P. Ferguson [1], Christopher Molloy [1], Priyakshi Kalita-de Croft [2], Sarah A. Hayes [3], Amanda Hudson [3], Emily Colvin [3], Hannah Kamitakahara [3], Rozelle Harvie [3], Csilla Hasovits [3], Tashbib Khan [4], Pascal H.G. Duijf [1,5,6,7,8], Viive M. Howell [3], Yaowu He [4], Emma Bolderson [1], John D. Hooper [4], Sunil R. Lakhani [2], Derek J. Richard [1], Kenneth J. O'Byrne [1,9,*] and Mark N. Adams [1,*]

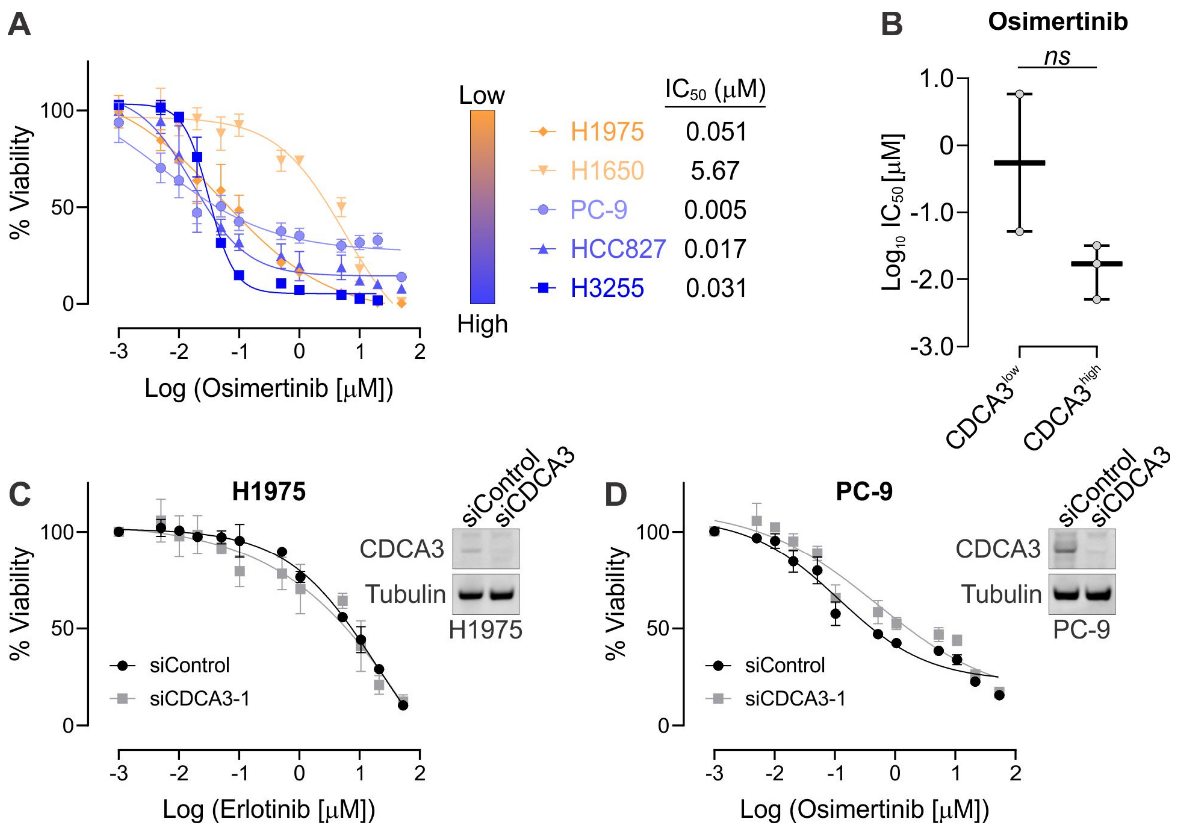

Figure S1. (**A**) *Left panel*, Dose response curves for five EGFR mutant NSCLC cell lines treated with escalating doses of osimertinib. Cells were treated with osimertinib for 72 hours before assessing cell viability. *Right panel*, Cell lines ranked by CDCA3 protein levels where osimertinib potency values (IC_{50}) were calculated using GraphPad Prism and listed for each cell line. n = 4. (**B**) Box and whisker plot showing osimertinib potency (log IC_{50} value) in $CDCA3^{low}$ and $CDCA3^{high}$ EGFR mutant cell lines (unpaired Student's *t* test, ns = not significant). (**C**) *Left panel*, Dose response curves for control (siControl) or CDCA3 depleted (siCDCA3) H1975 cells treated with escalating doses of erlotinib for 72 hours before assessing cell viability. *Right panel*, Endogenous CDCA3 western blot analysis from lysates of control or CDCA3 depleted H1975 cells. Tubulin was used as loading control. (**D**) *Left panel*, Dose response curves for control (siControl) or CDCA3 depleted (siCDCA3) PC-9 cells treated with escalating doses of osimertinib for 72 hours before assessing cell viability. *Right panel*, Endogenous CDCA3 western blot analysis from lysates of control or CDCA3 depleted PC-9 cells. Tubulin was used as loading control.

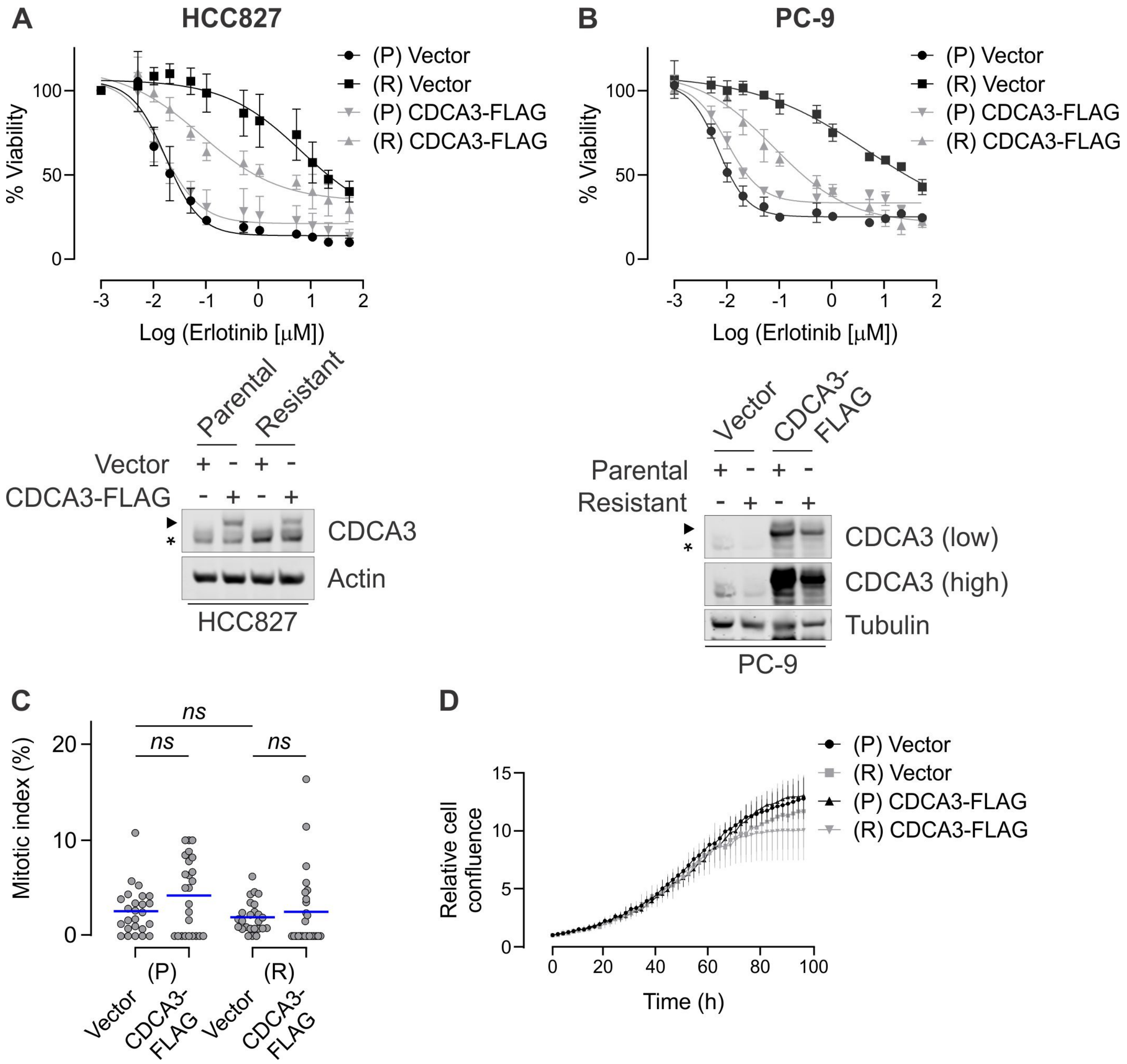

Figure S2. (**A-B**) *Top panels*, Dose response curves for vector transfected or ectopic CDCA3-FLAG overexpression in isogenic parental and resistant (**A**) HCC827 and (**B**) PC-9 cells treated with escalating doses of erlotinib for 72 hours before assessing cell viability. (**A-B**) *Bottom panels*, Western blot analysis of lysates from (**A**) HCC827 and (**B**) PC-9 isogenic parental and TKI resistant cells transfected with empty vector or CDCA3-FLAG. CDCA3 western blot analysis was performed to detect ectopic (*arrowhead*) and endogenous (*asterisk)* CDCA3. Tubulin was used as a loading control. (**C**) Beeswarm plot showing the mitotic index

determined by histone H3 pS10 staining and high throughput immunofluorescence microscopy of vector transfected or CDCA3-FLAG ectopically expressing HCC827 isogenic parental and TKI resistant cells. Data points represent an average percentage of mitotic nuclei per field of view from a minimum of 1100 nuclei (n = 23 fields total). Blue lines indicate median values. ns = not significant. (**D**) Cellular proliferation analysis of vector transfected or CDCA3-FLAG ectopically expressing HCC827 isogenic parental and TKI resistant cells over 96 h using the Incucyte Zoom live cell imaging system.
